# Driving pressure-guided ventilation improves homogeneity in lung gas distribution for gynecological laparoscopy: a randomized controlled trial

**DOI:** 10.1038/s41598-022-26144-8

**Published:** 2022-12-15

**Authors:** Wei Zhang, Feifei Liu, Zhanqi Zhao, Chunqing Shao, Xin Xu, Jiajia Ma, Ruquan Han

**Affiliations:** 1 Department of Anesthesiology, Beijing Tiantan Hospital, Capital Medical University, Beijing, 100070 People’s Republic of China; 2 Department of Anesthesiology, Beijing Fangshan District Liangxiang Hospital, Beijing, 102400 People’s Republic of China; 3 Department of Biomedical Engineering, Fourth Military Medical University, Xi’an, 710000 People’s Republic of China; 4 Institute of Technical Medicine, Furtwangen University, Villingen-Schwenningen, Germany; 5 Department of Clinical Diagnosis and Laboratory, Beijing Tiantan Hospital, Capital Medical University, Beijing, 100070 People’s Republic of China

**Keywords:** Randomized controlled trials, Outcomes research

## Abstract

To investigate whether driving pressure–guided ventilation could contribute to a more homogeneous distribution in the lung for gynecological laparoscopy. Chinese patients were randomized, after pneumoperitoneum, to receive either positive end expiratory pressure (PEEP) of 5 cm H_2_O (control group), or individualized PEEP producing the lowest driving pressure (titration group). Ventilation homogeneity is quantified as the global inhomogeneity (GI) index based on electrical impedance tomography, with a lower index implying more homogeneous ventilation. The perioperative arterial oxygenation index and respiratory system mechanics were also recorded. Blood samples were collected for lung injury biomarkers including interleukin-10, neutrophil elastase, and Clara Cell protein-16. A total of 48 patients were included for analysis. We observed a significant increase in the GI index immediately after tracheal extubation compared to preinduction in the control group (*p* = 0.040) but not in the titration group (*p* = 0.279). Furthermore, the GI index was obviously lower in the titration group than in the control group [0.390 (0.066) vs 0.460 (0.074), *p* = 0.0012]. The oxygenation index and respiratory compliance were significantly higher in the titration group than in the control group. No significant differences in biomarkers or hemodynamics were detected between the two groups. Driving pressure–guided PEEP led to more homogeneous ventilation, as well as improved gas exchange and respiratory compliance for patients undergoing gynecological laparoscopy.

**Trial Registration**: ClinicalTrials.gov NCT04374162; first registration on 05/05/2020.

## Introduction

Laparoscopy is preferred for gynecological surgery. However, pneumoperitoneum (PNP) and a steep Trendelenburg position (T-position) impose adverse effects on the respiratory system^1^, which lead to reduced homogeneity in gas distribution, impair gas exchange, and contribute to postoperative pulmonary complications (PPCs).

Lung protection ventilation (LPV) mitigates iatrogenic injury in previously healthy lungs to reduce the incidence of PPCs^2^. In addition to a lower tidal volume (VT), the positive end-expiratory pressure (PEEP) should initially be set to 5 cm H_2_O and individualized thereafter to minimize atelectasis and/or overdistention^3^. However, little is known about how to set the optimal PEEP under elevated intra-abdominal pressure in laparoscopy. Currently, multiple options for PEEP titration have been tried, for example, by pulmonary compliance^4^, intraabdominal pressure^5^ or by using electrical impedance tomography (EIT)^6^, but with inconsistent data.

Driving pressure (DP), calculated as [airway plateau pressure (Pplat)—PEEP]^7^, is the pressure needed for alveolar opening under controlled or assisted ventilation. For patients with or without healthy lungs, DP is more closely related to PPCs or survival than VT or PEEP^8,9^. Therefore, the “lowest DP”-based ventilation has been proposed to be a new direction, which has already been demonstrated in thoracic and abdominal surgeries, but not in gynecological laparoscopy^10,11^.

The effects of the ventilation protocol on lung outcome should be evaluated by imaging the recruitable lung volume for gas exchange. Thoracic electrical impedance tomography (EIT) is a functional radiation-free and noninvasive imaging technique at bedside, by which the dynamic changes in aeration distribution can be successfully visualized and evaluated^12^. It has been successfully validated by CT scans^13^ and safely used in both adult and pediatric patients^14,15^. The global inhomogeneity (GI) index is an EIT-based numeri value that explores homogeneity in the VT distribution. A higher GI implies more heterogeneous spatial ventilation in the lung^16^.

In this study, we hypothesized that DP-guided PEEP favors homogeneous ventilation measured by the GI index. Global parameters for lung function, such as oxygenation and respiratory mechanics, and lung injury biomarkers, such as the anti-inflammatory factor interleukin-10 (IL-10), the barotrauma indicator neutrophil elastase (NE), and the atelectrauma indicator Clara Cell protein-16 (CCP-16)^17^, were the secondary outcomes.

## Methods

### Ethical

The randomized, parallel-group, patient- and outcome assessor blinded trial was conducted at a single institution in accordance with the Declaration of Helsinki. This study was approved by Ethics Committee of Chinese Clinical Trial Registry (ChiECRCT20200112; May 2020) and registered before patient recruitment at clinicaltrials.gov (NCT04374162; 05/05/2020). Written informed consent was obtained from all participants one day prior to surgery and randomization. The study adhered to the applicable Consolidated Standards of Reporting Trials (CONSORT) guidelines and conformed to the Enhancing the QUAlity and Transparency Of health Research (EQUATOR) network guidelines.

### Patient selection

The inclusion criteria were: (1) patients 18 to 80 years old; (2) patients scheduled for elective gynecological laparoscopy in T-position; (3) mechanical ventilation of > 2 h; (4) expected postoperative extubation in the operation room. The exclusion criteria were ASA Physical Status of IV or V, mechanical ventilation of > 1 h within the last 2 weeks before surgery, BMI ≥ 35 kg∙m^−2^, respiratory diseases, emergency surgery, severe heart failure (cardiac index less than 1.8 L∙min^−1^∙m^−2^), progressive neuromuscular illness, pregnancy, refusal to participate and contradicted to EIT scan. Drop-out criteria included surgery type changed to open techniques and MAP dropped to below 55 mmHg.

### Randomization and blinding

Randomization was conducted by computer-generated random number allocation sealed in an opaque envelope. Eligible patients were randomized to two groups within 24 h before surgery by designated staff, with an allocation ratio of 1:1. The attending anesthesiologist in charge of the intervention was aware of the group assignment. Chest EIT was performed by a specialized technician and analyzed by a researcher. Data collection in the postoperative period and off-line data analysis were performed blindly.

### Anesthesia

A radial artery was cannulated for blood gas analysis and continuous blood pressure monitoring as a routine procedure for laparoscopy in our institution. A sample of venous blood for measurement of biomarkers was also obtained. All patients were preoxygenated with FiO_2_ > 0.8 and received routine anesthesia induction with intravenous sufentanil (2-3 μg∙kg^−1^), propofol (2-3 mg∙kg^−1^) and rocuronium (0.6 mg∙kg^−1^). Thereafter, anesthesia was maintained with sevoflurane (0.4 MAC) and propofol (3-4 mg∙kg^−1^∙h^−1^) to maintain bispectral index values of 40-60. MAP was maintained between ± 20% of the baseline value. Intraoperative analgesia was provided with continuous remifentanil infusion (0.05-0.2 μg∙kg^−1^∙min^−1^) and additional sufentanil if required. Rocuronium was repeated if needed. Ondansetron (8 mg) and tramadol (1.5 mg∙kg^−1^) were infused 15 min before the end of surgery and residual neuromuscular block was antagonized with neostigmine 0.04 mg∙kg^−1^ and atropine 0.02 mg∙kg^−1^ after spontaneous ventilation recovery postoperatively. Postoperative pain was controlled under 3 visual analog scores (VAS; 0: no pain; 10: pain as bad as it could be, or worst pain). All patients were transferred to postanesthesia care unit (PACU) after successful extubation and monitored for at least 1 h. The follow-up lasted 3 days postoperatively.

### Intervention

Eligible patients were randomly assigned to the control group or the titration group. The ventilation protocol consisted of volume-controlled mechanical ventilation (Datex Ohmeda S/5 Advance, General Electric Healthcare, Helsinki, Finland) at a VT of 8 ml∙kg^−1^ per predicted body weight, fresh gas of 2 L∙min^−1^, FiO_2_ = 0.4 (elevated if SpO_2_ < 94%), inspiratory to expiratory ratio of 1:2, and a respiratory rate adjusted to normocapnia (PaCO_2_ between 35 and 45 mmHg). Ventilation parameters, such as Pplat, peak pressure (Ppeak) and compliance, were derived from the same anesthetic machine. In the titration group, 10 min after PNP (intraabdominal pressure 14 mmHg) and 30° T-position, PEEP was increased stepwise by 1 cmH_2_O from the initial 5 cmH_2_O until reaching 15 cmH_2_O. Each level was maintained for 10 respiratory cycles, and in the last cycle, DP calculated as (Pplat-PEEP) was recorded. Then the level producing the lowest DP was identified as “individualized PEEP” and maintained until deflation of PNP at the end of the operation. The titration lasted no more than 10 min. If Pplat achieved more than 30 cmH_2_O, it was terminated in advance. In the control group, PEEP was fixed at 5 cmH_2_O throughout the whole ventilation. It is reported that intrinsic PEEP (PEEPi) results from delayed lung emptying in a wide range of respiratory conditions^18^, which have been excluded from this study, so PEEPi has no important clinical consequences here. However, if we identified the presence of PEEPi by real-time airflow and airway pressure vs time waveforms at the point of end-expiration, we recorded it.

### Outcomes

The primary outcome was the GI value immediately after extubation. Thoracic EIT (PulmoVista 500, Draeger Medical, Lübeck, Germany) was performed as described previously^13^ by a trained technician. Briefly, the silicon belt with 16 electrodes was placed around thorax circumference at the fifth intercostal space, with another reference electrode on the abdomen. The position of the silicon belt was marked at the skin for the second measurement immediately after extubation. The dynamic changes in aeration distribution can be visualized and measured by calculating the impedance changes during breathing cycles^12^. On this basis, the GI index was calculated offline using customized software and a lower index implied better postoperative lung recovery. Multiple studies in both the laboratory and clinic have supported EIT as a unique standard device of simplicity, efficacy, and safety^15^.

The secondary outcomes included oxygenation index (OI), DP, respiratory system compliance, plasma concentrations of biomarkers, hemodynamics perioperatively and PPCs within 3 days postoperatively. Arterial blood gas was tested (ABL 800, Radiometer, Copenhagen, Denmark) for OI as PaO_2_/FiO_2_. Venous blood samples collected in EDTA vials were centrifuged at 3,000 rpm for 10 min and stored in aliquots at − 80 °C for analysis. IL-10, CCP-16 and NE were measured using a Human Interleukin 10 ELISA Kit (Cat#CSB-E04593 h; CUSABIO, China), Human Clara cell protein ELISA Kit (Cat# CSB-E08680 h; CUSABIO, China) and Human elastase 2, neutrophil ELISA kit (Cat# CSB-EL007587 HU; CUSABIO, China), respectively. PPCs were evaluated with Melbourne Group Scale version 2^19^ (Appendix Table 1).

**Table 1:** Demographics and surgery characteristics.

	The control group (n = 24)	The titration group (n = 24)
Age; yr	38.1 (11.2)	43.8 (9.5)
Height; cm	163 (4.6)	161 (6.4)
Weight; kg	64.6 (11.9)	61.6 (10.0)
BMI; kg m^−2^	24.4 (4.0)	23.7 (2.8)
ASA, II, n (%)	24 (100)	22 (92)
III, n (%)	0 (0)	2 (8)
Hemoglobin, g dL^−1^	120 (18.8)	116 (18.5)
Hypertension, n (%)	1 (4)	4 (17)
Diabetes, n (%)	1 (4)	0 (0)
Anemia, n (%)	1 (4)	1 (4)
Heart disease, n (%)	0 (0)	1 (4)
Tidal volume; mL	440 (35)	430 (40)
RR; breaths min^−1^	13 (1)	13 (1)
Intraoperative fluid input; mL	1580 (680)	1550 (480)
Intraoperative bleeding; mL	110 (250)	120 (180)
Intraoperative fluid output; mL	480 (400)	600 (340)
Duration of operation; min	139 (55)	147 (65)
Duration of ventilation; min	180 (68)	197 (56)
Vasoactive drugs, n (%)	5 (21)	2 (8)
**Type of surgery, n (%)**		
Oophorectomy	11 (46)	1 (4)
Hysterectomy	5 (21)	14 (58)
Adnexectomy	2 (8)	2 (8)
Salpingoplasty	2 (8)	1 (4)
Myomectomy	2 (8)	2 (8)
Uterus repair	2 (8)	4 (17)

Values are the mean (SD) or number (%). *RR* respiratory rate; *VAS* visual analog scale; *PPCs* postoperative pulmonary complications.

### Statistical analysis

We attempted to detect a difference of 0.1 in GI between the two groups according to a previous study^20^, with an alpha level of 0.05 and an SD of 10% using an independent t test at a power of 90%. Allowing for a dropout rate of 5%, the sample was required to be 24 patients in each group. GraphPad Prism 8.0 (GraphPad software, USA) was used for statistical analyses. The Kolmogorov–Smirnov test was used to check for a normal distribution. Continuous variables with normal distribution are presented as mean (SD). Nonnormally distributed data are presented as the median (IQR [range]). Categorical variables were reported as the number (proportion) of patients. Two-way ANOVA followed by Tukey’s multiple comparison was conducted to evaluate the effects of group, time, and the interaction on GI, OI, respiratory mechanics and hemodynamic variables. Biomarkers were statistically compared within each group (at different time points) using a paired Student’s t test and between groups using an independent t test. Chi-square analysis or Fisher’s exact test were conducted for categorical variables in comparison between two groups. The Mann–Whitney test was conducted for nonnormally distributed data. We judged a *p* value of less than 0.05 to be significant for all tests.

## Results

**Figure 1:**
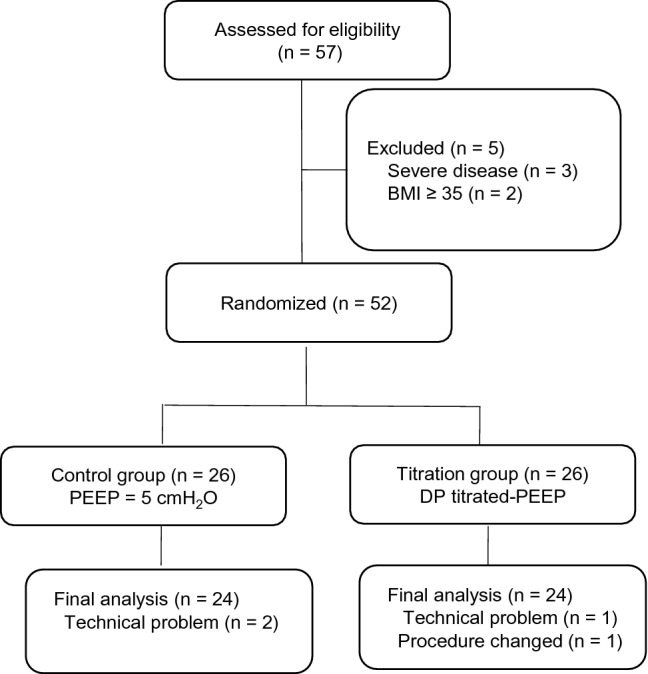
Flow diagram of the study. PEEP, positive end-expiratory pressure. *DP* driving pressure.

From May 2020 to February 2021, 57 patients undergoing elective laparoscopic gynecological surgery (Fig. 1) were screened. Five patients were excluded, and 52 patients were randomized into two groups and received the intended interventions. Two patients in the control group and one in the titration group were excluded due to technical problems in EIT. Additionally, one surgery was switched to an open abdomen procedure in the titration group. Finally, 24 patients in each group were analyzed. The demographic and clinical variables did not differ between groups, and no significant differences were observed in terms of VT, respiratory rate, fluid balance or ventilation duration (Table 1).

**Figure 2:**
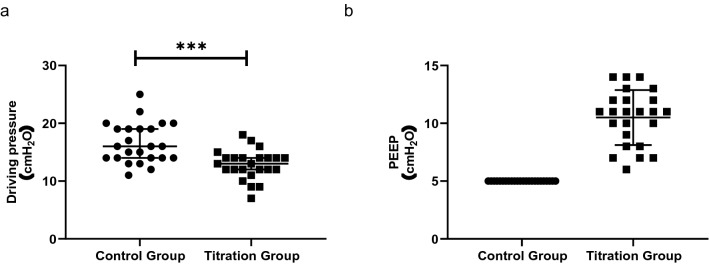
Distribution plot for driving pressure (**a**) and PEEP level (**b**). Lines indicate median with IQR. *PEEP* positive end-expiratory pressure. ***p < 0.001.

As seen from the distribution in Fig. 2, compared to the fixed PEEP of 5 cmH_2_O in the control group (Fig. 2b), we obtained an individualized PEEP in the titration group with a median (IQR [range]) of 11 (8-12 [6-14]) cmH_2_O, which led to a significantly lower DP of 13 (12-14 [7-18]) cmH_2_O than in the control group of 16 (14-19 [11-25]) cmH_2_O (*p* < 0.001, Fig. 2a).

**Figure 3:**
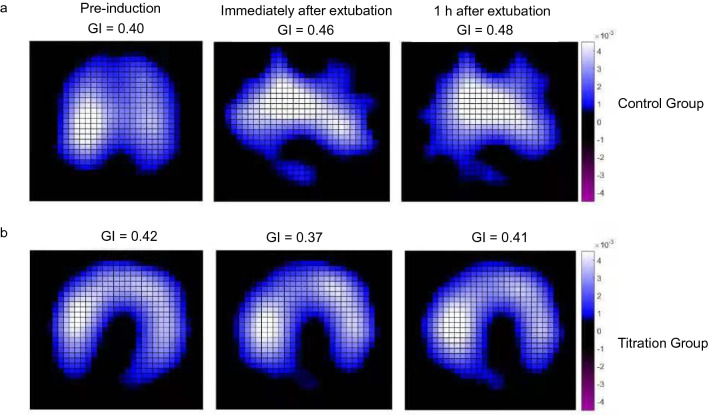
Representative images showing tidal variation measured by electrical impedance tomography at different time points for patients from (**a**) the control group and (**b**) the titration group. *GI* the global inhomogeneity index.

**Figure 4:**
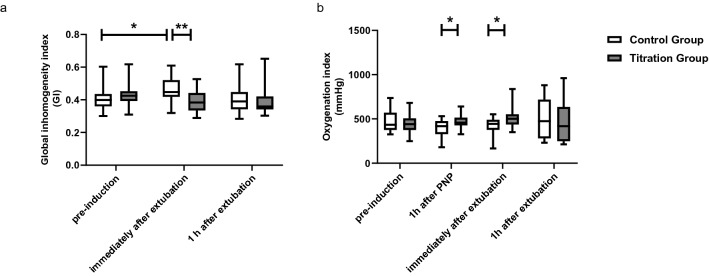
Box-whisker plots for the global inhomogeneity index (**a**) and oxygenation index (**b**) at different time points. Line at median, top of the box at the 75th percentile, bottom of the box at the 25th percentile, whiskers at the highest and lowest values. The oxygenation index was calculated as PaO_2_/FiO_2_. PNP, pneumoperitoneum. *p < 0.05; **p < 0.01.

Representative EIT images before and after surgery are shown in Fig. 3. In the control group, there was an increase of 15% in GI immediately after extubation compared to preinduction (*p* = 0.04), implying an unfavorable gas redistribution in the lung after laparoscopy. Furthermore, this value was obviously higher than that in the titration group [0.460 (0.074) vs 0.390 (0.066), *p* = 0.001] immediately after extubation, suggesting a more homogeneous distribution of gas by ventilation with DP-guided PEEP (Fig. 4a). Regarding the global lung function, OI was significantly higher in the titration group than in the control group 1 h after PNP [460 (73) vs 398 (99) mmHg, *p* = 0.02)], as well as immediately after extubation [515 (123) vs 429 (95) mmHg, *p* = 0.03)] (Fig. 4b).

There was an obvious decline in static compliance 10 min after PNP in both groups (*p* < 0.0001 in both groups), with significant differences between the groups evolving along distinct ventilation protocols (*p* = 0.02). Although both the Ppeak and Pplat were higher in the titration group 1 h after PNP (*p* = 0.02 and *p* = 0.01 vs Control group, respectively) and 10 min after deflation of PNP (*p* = 0.03 and *p* = 0.04 vs Control group, respectively), these levels were within the safe limit. PaCO_2_ and pH did not show any difference between the two groups perioperatively (Table 2).

**Table 2:** Ventilatory mechanics and gas analysis.

Characteristics	Control	Titration	*p*		
	(n = 24)	(n = 24)	Time	Group	Inter
**Compliance (mL cmH**_**2**_**O**^−**1**^**)**			< 0.001	0.02	< 0.001
immediately after intubation	62.5(10.4)	60.8(9.3)			
10 min after PNP	32.3(6.6)	29.3(5.4)			
1 h after PNP	29.4(6.1)	36.7(8.3)**			
10 min after deflation of PNP	43.0(11.8)	52.5(11.1)*			
end of surgery	44.1(10.7)	55.1(10.3)**			
**Ppeak (cmH**_**2**_**O)**			< 0.001	0.08	< 0.001
immediately after intubation	14(3.9)	14.5(2.6)			
10 min after PNP	21.5(3.8)	22.1(2.6)			
1 h after PNP	23(4.6)	26.3(2.5)*			
10 min after deflation of PNP	17.7(3.1)	20.5(3.5)*			
end of surgery	17.8(3.2)	17.1(3.8)			
**Pplat (cmH**_**2**_**O)**			< 0.001	0.06	0.01
immediately after intubation	11.5(4.1)	12.2(2.8)			
10 min after PNP	19.5(4.0)	20.0(3.0)			
1 h after PNP	21.5(4.0)	24.5(2.1)*			
10 min after deflation of PNP	15.2(3.2)	17.9(3.7)*			
end of surgery	14.9(2.8)	14.9(3.7)			
**PaCO**_**2**_			< 0.001	0.89	0.39
immediately after intubation	35.9(4.1)	34.0(4.1)			
1 h after PNP	41.9(4.0)	41.9(5.5)			
immediately after extubation	45.0(7.2)	46.3(6.0)			
1 h after extubation	39.4(4.3)	40.1(4.5)			
**pH**			< 0.001	0.05	0.33
immediately after intubation	7.41(0.028)	7.43(0.039)			
1 h after PNP	7.34(0.042)	7.36(0.047)			
immediately after extubation	7.32(0.045)	7.32(0.046)			
1 h after extubation	7.35(0.039)	7.36(0.045)			

Values are the mean (SD) or number (%). Values are the mean (SD). *Ppeak* airway peak pressure; *Pplat* airway plateau pressure; *PNP* pneumoperitoneum. *p < 0.05; **p < 0.01 compared to the control group.

**Figure 5:**
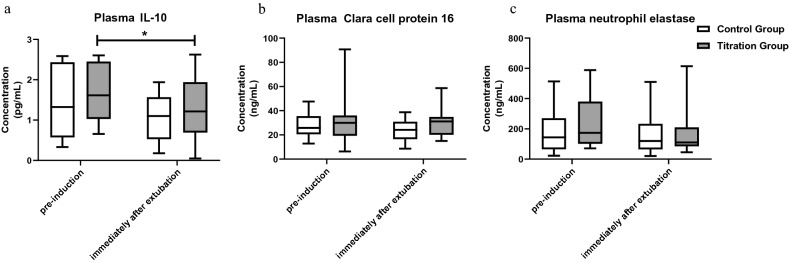
Box-whisker plots for plasma concentrations of IL-10 (**a**), CC16 (**b**), and NE (**c**) at different time points. Line at median, top of the box at the 75th percentile, bottom of the box at the 25th percentile, whiskers at the highest and lowest values. *IL-10* interleukin-10. *CCP-16* Clara cell protein 16. *NE* neutrophil elastase. *p < 0.05.

CCP-16 and NE did not change over time in either group, and no differences were found between the two groups immediately after extubation (*p* = 0.10 for CCP-16 and *p* = 0.99 for NE, Fig. 5). The plasma concentration of IL-10 from patients in the titration group significantly decreased after mechanical ventilation, compared to preinduction (*p* = 0.04) but not in the control group (*p* = 0.25). There were no significant differences between the two groups immediately after extubation. Regarding the hemodynamic parameters, both MAP and HR were comparable throughout surgery in the two groups (Appendix Fig. 1). We observed no differences concerning demands for vasoactive drugs (*p* = 0.42). At the 3-day follow-up, one patient in the control group and two in the titration group reported a VAS pain score of > 3 (*p* > 0.99), and no PPCs defined as the Melbourne Group Scale of at least 4 occurred in either group.

## Discussion

The main findings of this randomized controlled study were: (i) individualized PEEP guided by DP improved ventilation distribution, gas exchange and pulmonary mechanics compared to a fixed PEEP of 5 cm H_2_O during gynecological laparoscopy; (ii) there were no significant differences in hemodynamic parameters and blood gas analysis in both groups; (iii) no major adverse events occurred throughout the study; (iv) the benefits of DP-guided ventilation did not disappear after extubation. So DP could be a promising target for lung-protection ventilation strategies to reduce PPCs, and individualized PEEP titration aimed at the lowest DP was proposed as an effective and time-efficient approach in clinical utility.

PPCs remain a worldwide healthcare problem especially for abdominal surgery of at least 2 h duration^21^. A reduction of 30% in the mean vital capacity percentage has been reported after laparoscopic prostatectomy in nonobese patients^22^. DP, the pressure required to open the alveoli, was inversely related to compliance and orthodromicly related to VT (intrinsically normalized to functional lung size)^23^. An international consensus on lung protection has also recommended avoiding increase in DP^3^. A recent retrospective cohort analysis^24^, which included 2034 patients undergoing abdominal surgery, concluded that DP was significantly associated with PPCs and intraoperative adverse events in both open and closed abdominal surgeries. The association was stronger in closed abdominal surgery than in open abdominal surgery (risk ratio (RR), 1.11 [95% CI 1.10 to 1.20], *p* < 0.001). Therefore, titrating PEEP to obtain the lowest DP could be an effective preventive strategy against PPCs, especially for closed abdominal surgery such as laparoscopy.

Accordingly, recent prospective studies demonstrated that PEEP optimization guided by DP is associated with a lower incidence of PPCs in both thoracic and abdominal surgery^2,25^. For elderly patients undergoing laparoscopic surgery^26^, DP-guided individualized PEEP has reduced lung atelectasis at the end of surgery and 15 min after admission to PACU. The intraoperative respiratory mechanics have also been improved. Meanwhile, the study indicated that ventilation strategy with a fixed PEEP of 6 cmH_2_O was not superior to that with zero PEEP in reducing postoperative pulmonary atelectasis. All these are supportive of our results. A DP higher than 16 cmH_2_O has been associated with an increased risk of PPCs for noncardiac surgeries^27^. In our study, 22 patients (22/24) in the titration group yielded a DP value of no more than 16 cm H_2_O compared to the control group of only 14 patients (14/24, *p* = 0.01) (Fig. 2a). The lower DP in the titration group led to an improved gas distribution and exchange in the lung, suggesting that it is a promising marker for subsequent lung injury. We also observed a median DP difference of only 3 cmH_2_O between groups. Previous studies have supported this by showing that each 1 cmH_2_O increase in DP was associated with worse respiratory outcomes for ARDS patients, as well as surgical people with healthy lungs^9,10^. All these results indicated that what matters is the individualization of ventilation but not the absolute data. The relatively higher DP discrepancy in our study (median 3 cmH_2_O) may be attributed to the added workload to counteract PNP and T-position, suggesting that DP-guided ventilation bears a stronger pathophysiological rationale for laparoscopy surgery.

In patients undergoing lower abdominal surgery, atelectasis and impaired arterial oxygenation were observed during mechanical ventilation, as well as the first postoperative days^28^. Utilizing synchrotron-based X-ray tomographic microscopy on isolated rat lungs, experimental data have estimated that a small global strain can lead to local strains up to four times as high in the alveolar wall of heterogeneous lungs^29^. The strain hotspots obviously tend to be at the thinnest regions of the alveolar walls, which seem to become overstretched. Furthermore, dynamic PET/CT imaging of [18F] fluoro-2-deoxy-d-glucose in piglets has provided new information that normally/poorly aerated regions are the primary targets of the inflammatory process accompanying early VILI, suggesting that tidal stretch is highest in these intermediate gravitational zones^30^. All these findings emphasize the importance of strategies capable of minimizing both collapse and hyperinflation, thus unloading the ventilated lung. In the presence of anesthesia-related atelectasis, DP scales the tidal volume in relation to the remaining ventilated lung size and the mechanical scenario created by PEEP, so it is important to individualize the ventilatory settings and achieve an optimum DP adapted to the size of the ventilated lung. In the present study, DP-guided PEEP led to a lower GI index immediately after extubation, suggesting that the heterogeneity caused by surgery and anesthesia was restored. Postoperative OI might be a potential target independently associated with PPCs and mortality^31^. Here, we observed a better perioperative OI in the titration group, which could be crucial in high-risk patients for PPCs even if no hypoxic events occurred in either group.

Individual titrated PEEP provides the optimum compromise to alleviate the heterogeneous ventilation distribution. In this study, the individualized PEEP values ranged between 6 and 14 cmH_2_O with a median of 11 cmH_2_O, strengthening that a fixed PEEP is not suitable due to the individual characteristics of the patient and surgery. Here, we chose increment titration, a similar approach that has been used in studies for thoracic surgery^10^. Increment titration is commonly used in both ARDS patients and surgical patients with healthy lungs and appears to be more time-efficient and easier for clinical utility^10,32,33^. Reasons for not applying RM include concern about hypoxemia and hemodynamic instability and the risk of airway secretions dislodged by RM distally^34^. However, a recent study showed a significant effect of an intensive alveolar recruitment as a step further in postoperative lung protection for vasoplegic patients after cardiac surgery without significant side effects^35^. Moreover, it has been illustrated that in an early ARDS patient, the dynamic compliance, oxygenation and reduction in DP appeared limited during the incremental phases, compared to the decremental phrase with the same PEEP levels, suggesting that DP can be further reduced by means of individual maximal lung recruitment and decremental PEEP titration, which may induce a more homogeneous distribution of transpulmonary pressures and a better gain in lung compliance^36^. This alternative approach has been supported in more recent studies for both healthy and injured lungs subjected to mechanical ventilation^37–39^.

We noted that group differences in GI and OI disappeared at 1 h after extubation, and no PPCs occurred in either group. It has been reported that the benefits of individual PEEP in GI, end-expiratory lung volume, and oxygenation vanish 2-6 h after extubation in both nonobese and obese patients undergoing laparoscopic surgery^40^. The ventilation strategy with DP-guided PEEP has not influenced the incidence of PPCs in elderly patients undergoing laparoscopic surgery. Together with our results, all these are consistent with the consensus of an international expert panel that the benefits of individualized PEEP during ventilation may disappear quickly after extubation^3^. Ventilation therefore exerts its effects on lung distribution and gas exchange mainly throughout the surgery and the immediate extubation phase. Thus, to propagate the obtained benefits of DP-guided ventilation to long-term outcomes, additional strategies are needed, such as early mobilization.

In this study, plasma indicators for ventilation-associated lung injury, including IL-10, CCP-16 and NE, displayed no differences between the two groups, consistent with the findings in healthy patients ventilated during surgery^17,41^. The negative findings were possibly due to patients’ normal pulmonary status, the short study duration, and the limited sensitivity of measurements. However, the plasma level of the anti-inflammatory cytokine IL-10 significantly decreased in the titration group after mechanical ventilation, as supported by Ioanna Korovesi’s study^42^, which found that IL-10 concentrations in the exhaled breath condensate of ventilated uninjured lungs decreased over time during PEEP (8 cm H_2_O) and tended to increase in the ZEEP (0 cm H_2_O) group.

Intraoperative hypotension was more frequent in patients with higher PEEP^43^. We observed no difference in hemodynamic parameters and vasopressor requirements, which could be explained by the relatively lower Ppeak preset within the safety limit (< 30 cmH_2_O) and the avoidance of RM. Additionally, intraoperative PaCO_2_ and pH did not differ between the two groups, indicating adequate ventilation delivered in the titration group despite higher PEEP.

There were several limitations in our study. First, the sample is limited to a single center and to relatively healthy and nonobese females with normal lung function. However, DP-guided ventilation implies a promising strategy in this study and encourages us to generalize it to a larger population, especially those at risk for PPCs. Second, the DP-targeting strategy should not be used in isolation, without considering other ventilatory variables such as VT and PaCO_2_, and clinical factors such as lung disease and hemodynamics^44^. In our study, these factors were comparable in the two groups. Third, for practical reasons, the GI index was not measured during PNP and therefore remains to be studied in a future trial. Fourth, we chose incremental PEEP titration instead of decremental titration in this study. However, an incremental PEEP trial results in variable end-inspiratory recruitment, which affects end-expiratory recruitment at any particular PEEP level. Similar effect could be derived from inspiratory pressure–volume loop^45^. The hysteresis of the pressure–volume curve results from variation of surface tension from inspiration to expiration, from stress relaxation, from the viscoelastic properties of the lung, from different lung volumes and lung history, from different degrees of inspiratory recruitment and expiratory derecruitment. Therefore, characteristics of a deflation pressure–volume curve (e.g. decremental PEEP trials) after recruitment maneuver, allow better determination of an optimum PEEP^37,46^. Whether the findings in the present study could be transferred directly to decremental PEEP trial requires further exploration.

## Conclusion

DP-targeted individualized PEEP results in more homogeneous ventilation immediately after extubation in patients undergoing gynecological laparoscopy, as well as improved oxygenation and respiratory compliance perioperatively.

## Supplementary Information

Supplementary Figure 1.

Supplementary Table 1.

## Data Availability

The datasets generated and/or analyzed during the current study are not publicly available, as the data also form part of an ongoing study. However, the datasets are available from the corresponding author upon reasonable request.
