# Supplementary material for: Driving pressure-guided ventilation improves homogeneity in lung gas distribution for gynecological laparoscopy: a randomized controlled trial
Source: Sci Rep. 2022 Dec 15;12:21687. doi: 10.1038/s41598-022-26144-8 (PMC9755264; doi:10.1038/s41598-022-26144-8)
Supplement: Supplementary file 1 — Supplementary Figure 1. [file 41598_2022_26144_MOESM1_ESM.pdf]

Appendix Figure 1

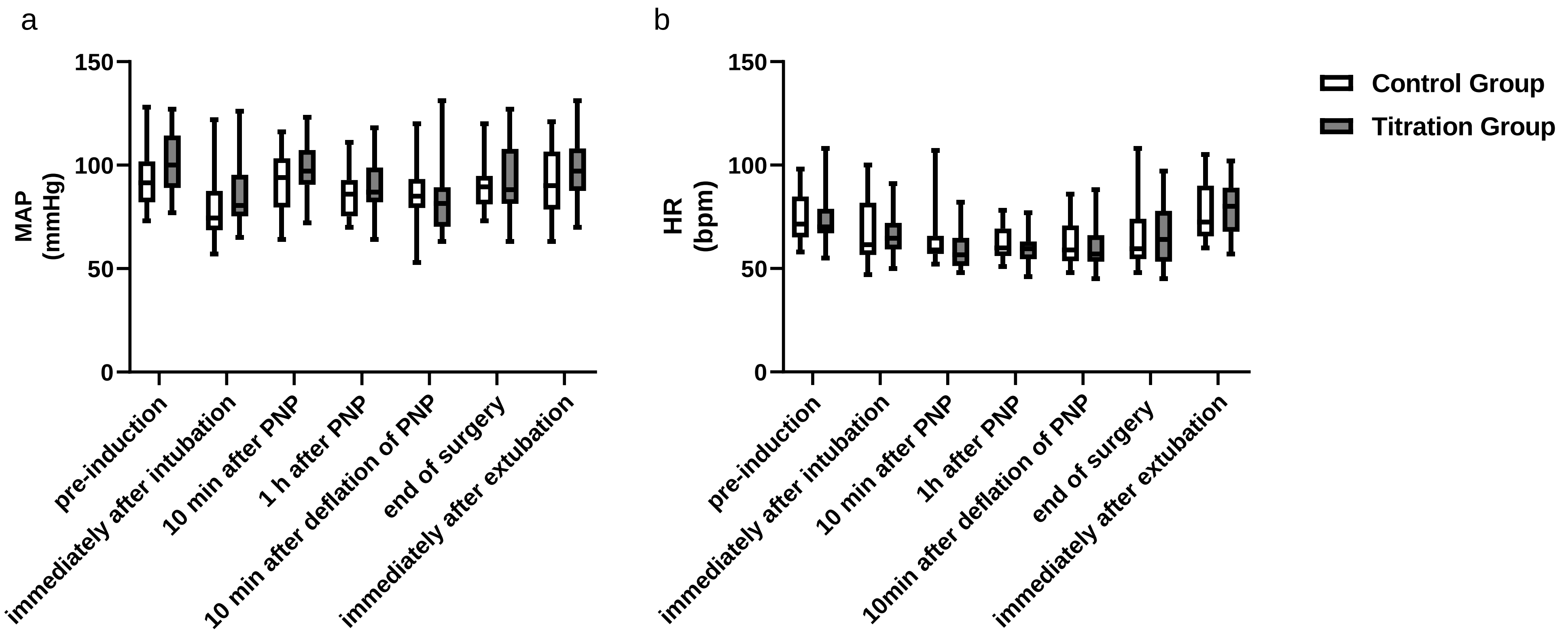

Box-whisker plots for MAP (a) and HR (b) at different time points. Line at median, top of the box at the 75th percentile, bottom of the box at the 25th percentile, whiskers at the highest and lowest values. MAP, mean arterial pressure. HR, heart rate.
