# Supplementary material for: Driving pressure-guided ventilation improves homogeneity in lung gas distribution for gynecological laparoscopy: a randomized controlled trial
Source: Sci Rep. 2022 Dec 15;12:21687. doi: 10.1038/s41598-022-26144-8 (PMC9755264; doi:10.1038/s41598-022-26144-8)
Supplement: Supplementary file 2 — Supplementary Table 1. [file 41598_2022_26144_MOESM2_ESM.docx]

Appendix Table 1

Melbourne Group Scale version 2

| Criteria* |
| --- |
| • Temperature >38°C  • White cell count >11.2 or use of respiratory antibiotics  • Physician diagnosis of pneumonia or chest infection  • Chest X-ray findings of atelectasis/consolidation  • Production of purulent (yellow/green) sputum different from preoperative sputum  • Positive results upon sputum microbiological analysis  • SpO2 <90% in ambient air  • Re-admission to or prolonged stay (>36 h) in the intensive care unit/high dependency unit for respiratory problems |

* A postoperative pulmonary complication is diagnosed if 4 or more of the 8 factors are present.
